# The effect of topical ketamine administration on the corneal epithelium repair

**DOI:** 10.1038/s41598-022-24639-y

**Published:** 2022-12-12

**Authors:** Mehdi Sanatkar, Zohre Nozarian, Fatemeh Bazvand, Parisa Abdi

**Affiliations:** 1 Anesthesiology, Critical Care and Pain Department, Farabi Eye Hospital, Tehran University of Medical Sciences, Tehran, Iran; 2 Pathology Department, Farabi Eye Hospital, Tehran University of Medical Sciences, Tehran, Iran; 3 Retina Department, Farabi Eye Hospital, Tehran University of Medical Sciences, Tehran, Iran; 4 Cornea Department, Farabi Eye Hospital, Tehran University of Medical Sciences, Tehran, Iran; 5 Translational Ophthalmology Research Center, Tehran University of Medical Sciences, Tehran, Iran

**Keywords:** Diseases, Neurology

## Abstract

The cornea is regarded as a sensitive organ to pain. Ketamine can effectively reduce postoperative neuropathic pain. We hypothesized that topical ketamine could mitigate postoperative corneal neuropathic pain. The aim of this study was to determine whether topical ketamine is safe for cornea and evaluate its effect on the repair procedure the damaged corneal tissue. Our study was performed on only the right eyes of 15 male rats. All animals underwent general anesthesia and the whole corneal epithelium was removed. All subjects were divided into two groups: group 1 (n = 8), one drop of ketamine, and group 2 (n = 7), one drop of 0.9% sodium chloride administered topically on the scraped cornea every 6 h for 7 days. The rats’ s cornea was carefully monitored daily for the size of epithelial defects under a microscope and was photographed. On the eighth day, the eyes were sent for pathological examination. The eyes were examined for the amount of inflammation, neovascularization, keratinization, epithelial thickness and Descemet's membrane pathologies. The epithelial defect has healed completely on the sixth day in all rats in both groups. There was no significant difference in the speed of complete recovery between the two groups. No significant difference was observed between the two groups in terms of inflammation grade, neovascularization grade, and epithelial thickness. Our study showed that topical ketamine had no significant effect on corneal wound healing in a rat animal model and could be used safely for the management of postoperative chronic ocular pain.

## Introduction

Pain is an unpleasant sensation and emotional experience that occurs following damage to nerve-ending fibers. Pain relief is of great importance in clinical practice. Pain consists of two general categories: nociceptive and neuropathic. Nociceptive pain is caused following damage to nociceptors and is accompanied by the intact somatosensory nervous system while neuropathic pain is created following damage or disease of the somatosensory nervous system^1^. The cornea is heavily innervated and is extremely sensitive to pain. It contains unmyelinated sensory fibers, and the density of these fibers is 300 to 600 times that of the dermal layer of skin. These sensory fibers transmit nociceptive input through ciliary nerves to the ophthalmic division of the trigeminal nerves. Injury or disease of corneal nerve fibers by peripheral and central sensitization can cause chronic neuropathic pain^2^. The nociceptors of the cornea are stimulated by various reasons, such as trauma, decreased tear volume, increase in tear osmolarity, and appearance of inflammatory cytokines causing irritation, discomfort, and dry-eye syndrome. Dry-eye syndrome and ocular neuropathic pain present with symptoms, such as persistent dysesthesia, hyperalgesia and allodynia^3^. Moreover, central sensitization triggers ocular neuropathic pain and is mostly resistant to traditional management. Previous studies identified the involvement of NMDA receptors in the pathogenesis of neuropathic pain in the peripheral and central nervous systems^4^. Researchers have found evidence of the presence of NMDA receptors in the peripheral somatic nerve fibers, spinal cord and supra-spinal nervous system, and the role of these receptors in central sensitization^5^. It has been found in earlier studies that an NMDA receptor antagonist such as ketamine can effectively reduce neuropathic pain in animal models and clinical trials^6^. The mechanism of action of ketamine in reducing neuropathic pain is explained by binding to the phencyclidine site of glutamate receptors^7^. Ketamine plays an inhibitory role on glutamate in the nervous system by the blocking of NMDA receptors and narrowing down the influx of calcium ions through these channels^8^. Previous studies have shown the role of ketamine in reducing postoperative pain in some experimental and clinical trial studies^9^. Koizuka et al. concluded that ketamine raises the hypersensitivity threshold of peripheral and central neurons in the postoperative pain model in rats^10^. We hypothesized that topical ketamine could mitigate postoperative corneal neuropathic pain. To the best of our knowledge, there was no study that evaluated the effect of topical ketamine on postoperative chronic corneal pain. Therefore, before starting the study in human subjects, it was necessary to first evaluate the safety of topical ketamine in corneal wound healing. Therefore, this study aimed to determine the safety of topical ketamine on the epithelium of cornea and evaluate the effect of topical ketamine on the repair process of damaged corneal tissue.

## Materials and methods

### Animal model of injury and treatment

The research ethics committee of laboratory animals, Tehran University of Medical Sciences, approved the study protocols (IR.TUMS.1401.007). This study was in accordance with relevant guidelines and regulations. All parts of methods were reported in accordance with ARRIVE guidelines. Our study was conducted on only the right eyes of 15 male rats, each weighing 200 g. All subjects were housed on a 12-h light and dark cycle in a temperature-controlled rack with easy access to water and food. Before starting the procedure, all animals underwent general anesthesia by intramuscular administration of ketamine (30 mg/kg) and xylazine (5 mg/kg) and for maintaining anesthesia, half of the initial doses of ketamine and xylazine were administered as needed. For the analgesia of corneal epithelium, one drop of 0.5% tetracaine HCL was administered only to the right eye. Then, after the instillation of a drop of 5% povidone-iodine, the whole corneal epithelium was removed. In addition, 20% dilute ethanol was applied to the corneal surface for 15 s, to facilitate epithelial removal, which was irrigated with a balanced salt solution. The corneal epithelium was then removed with a No. 15 surgical blade under an operating microscope. All subjects were divided into two groups and treated as follows: In group 1 (n = 8), one drop of ketamine (50 mg/ml) was administered topically on the scraped cornea every 6 h for 7 days. In group 2 (n = 7), one drop of 0.9% sodium chloride was administered topically every 6 h. A drop of chloramphenicol 0.5% was administered topically every 12 h in both groups to prevent microbial infections.

### Clinical examination

The condition of the rats’ cornea was carefully monitored daily for the size of epithelial defects under the microscope and photographed. Corneal epithelial defects were assessed after fluorescein vital staining with fluorescein strips. After staining, the size of the defect was measured using a caliper, and microscopic photographs were taken. Sizes of epithelial defects on photographs were measured by Image J software.

### Histological examination

Eight days after the procedure, all subjects underwent general anesthesia and then euthanized by injecting 100 mg/kg intravenous pentobarbital sodium, and the right eyes of the subjects were enucleated. Surgically removed eyes were fixed in 10% neutral buffered formalin immediately after enucleation for 24 h. Eyes were opened in the horizontal meridian to include both the optic nerve and pupil, and all eyes were processed and embedded in a paraffin block. Tissue sections were cut vertically through the optic disc at a thickness of five micrometers; therefore, the anterior portion of each section contained the cornea, and the posterior portion contained the optic disk. The tissue sections underwent hematoxylin and eosin staining and were examined by light microscopy. The eyes were examined for the amount of inflammation, neovascularization, keratinization, epithelial thickness and Descemet's membrane pathologies. The eyes were classified into 4 groups in terms of inflammation based on the average density of inflammatory cells in each power field (grade 0: no inflammation, grade 1: mild inflammation, grade 2: moderate inflammation, grade 3: severe inflammation)^11^ and into 4 groups in terms of corneal neovascularization (grade 0: No blood vessels visible in corneal stroma grade 1: < 25% of the corneal surface covered with developing network of blood vessels, grade 2: 26–75% of the corneal surface covered with developing network of blood vessels, grade 3: > 75% of the corneal surface with developed network of blood vessels)^12^. Epithelial thickness was measured by counting the epithelial cell layers. In addition, the presence or absence of keratinization on the corneal surface, pathologic changes in Descemet's membrane such as alterations in thickness, and the presence of rupture or folding were reported^13^.

### Statistical analysis

To describe data, mean, standard deviation, median and range were calculated. To compare outputs between the two groups, Mann–Whitney *U* test was run. In addition, the 95% confidence interval (95% CI) was considered for the mean difference of the two groups. Also, an interaction analysis within a linear mixed model was carried out to check the slope difference of decrease between two groups. In the last step, to compare the healing rate between the two (100% of heal), Log-rank test was employed and demonstrated this rate with Kaplan–Meier curve. All statistical analyses were performed by SPSS (IBM SPSS Statistics for Windows, Version 25.0. Armonk, NY: IBM Corp.) P-values less than 0.05 were considered statistically significant.

## Results

**Figure 1:**
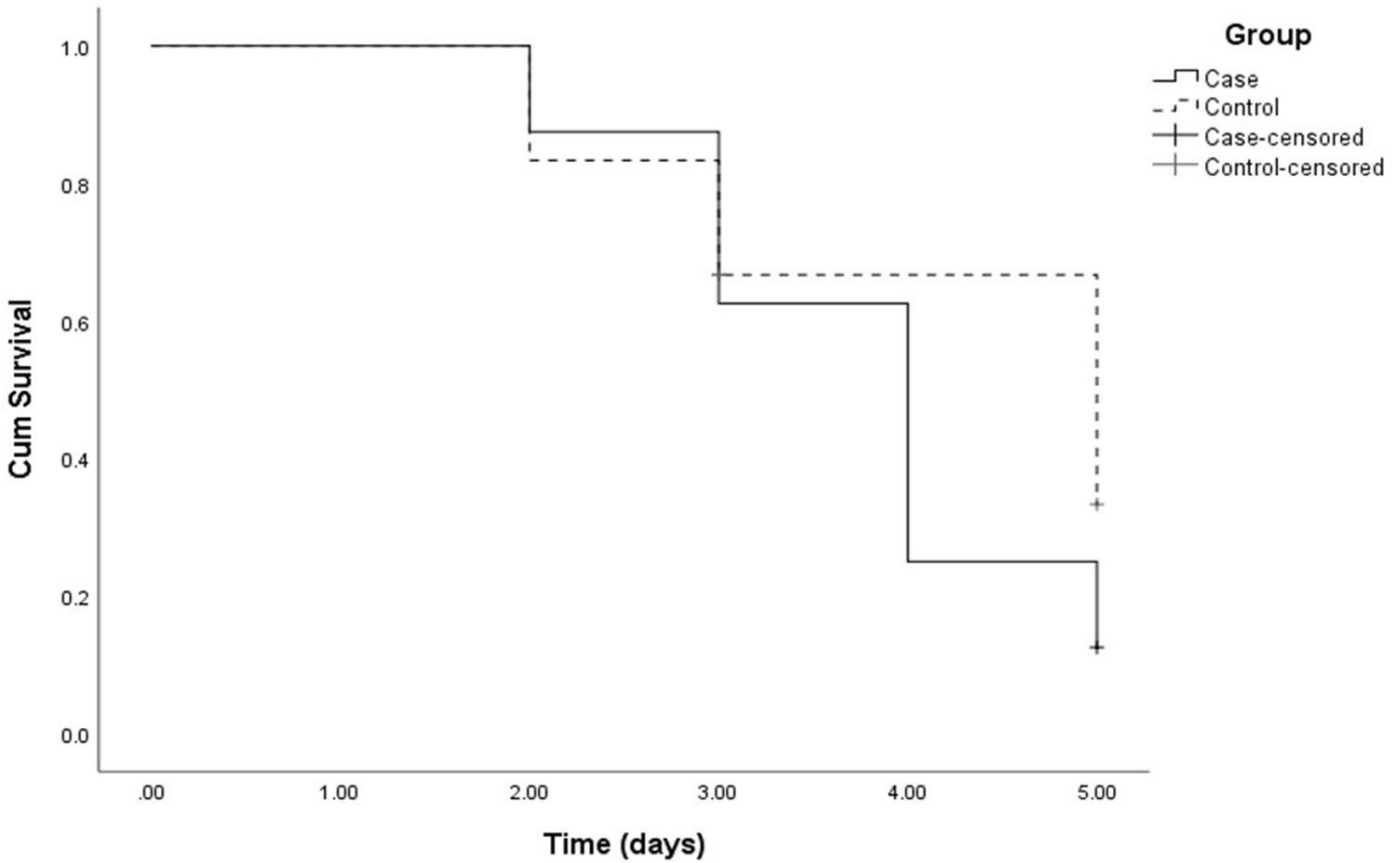
Healing rate (the speed of complete recovery) in two groups (P = 0.241, based on log-rank test).

**Figure 2:**
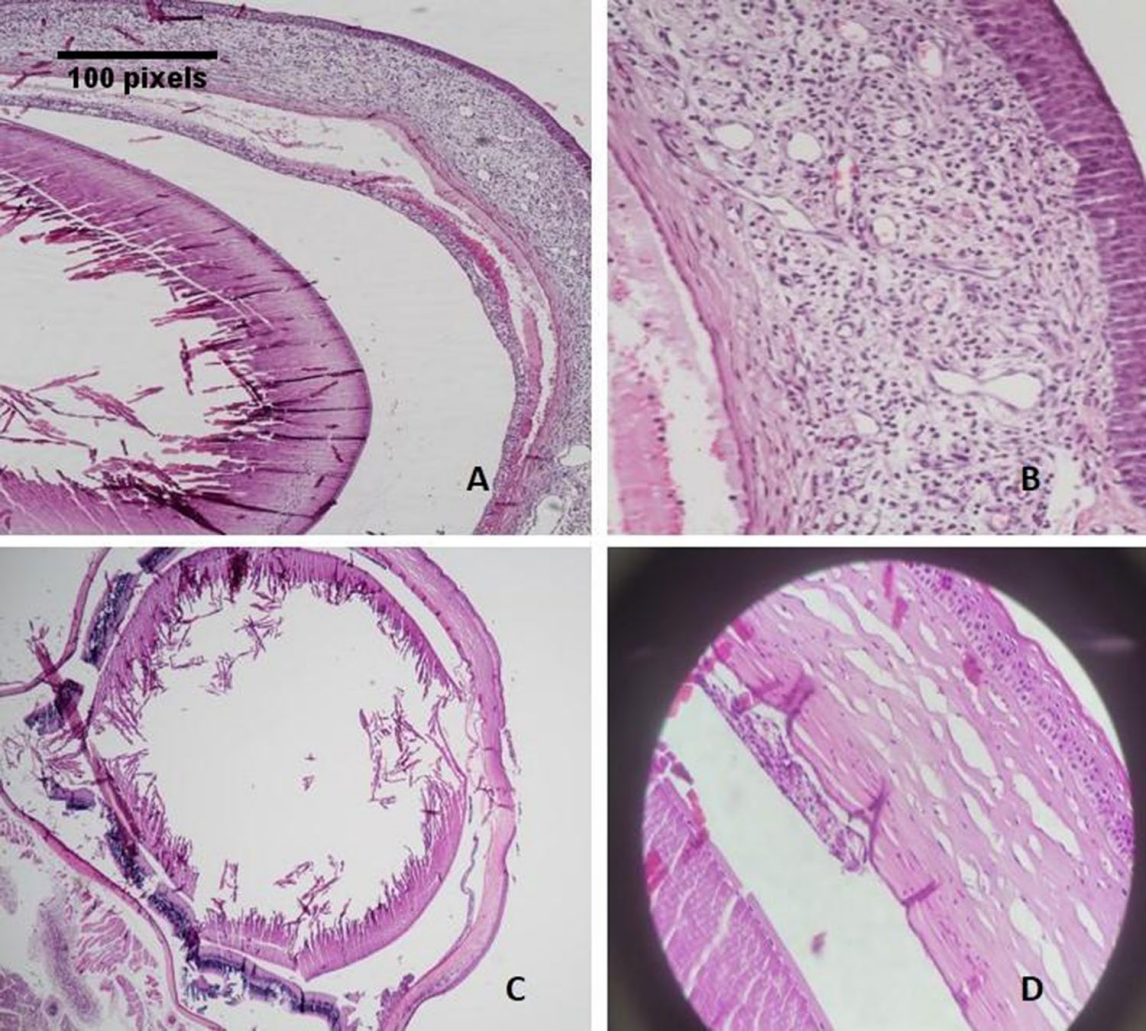
Globe (**A**) and cornea (**B**) sections related to case series. Globe (**C**) and cornea (**D**) sections related to control series.

**Figure 3:**
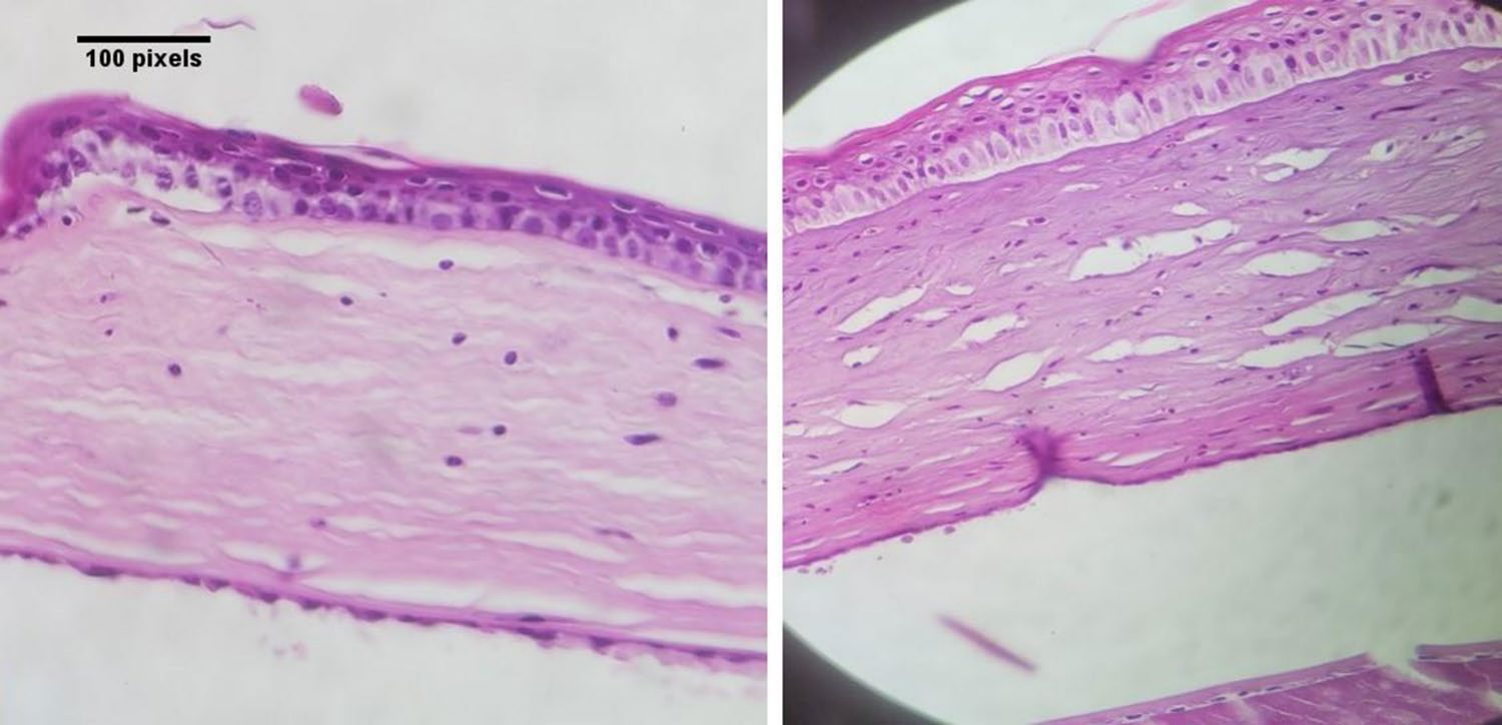
H/E-stained slides show edematous epithelium, mild infiltration of inflammatory cells in stroma, normal thickness of Descemet membrane lined by endothelium in a rat of ketamine group (right) and a rat in sodium chloride group (left).

**Figure 4:**
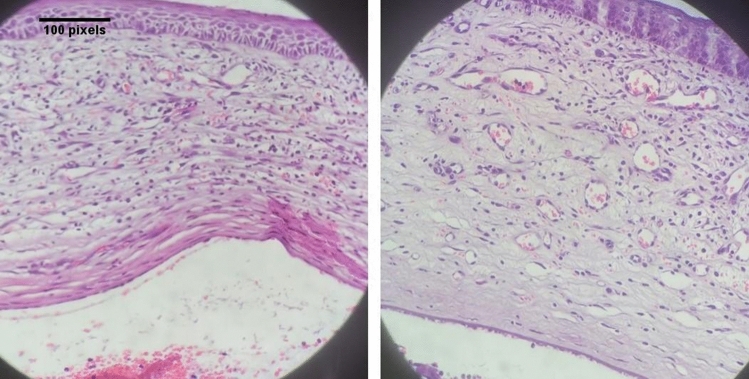
H/E-stained slides of both specimens show edematous epithelium, severe infiltration of inflammatory cells in stroma, severe neovascularization, normal Descemet membrane thickness lined by endothelium in one case in ketamine group (right) and one subject in sodium chloride group (left).

Eight rats in the ketamine group and seven rats in the control group were analyzed in terms of the size of the epithelial defect and the rate of reduction in the defect size. There was no significant difference between the two groups in terms of epithelial defect size reduction compared to day zero. The percentage reduction in size compared to day zero is shown in Table 1. Also, it is shown that the epithelial defect has healed completely on the sixth day in all the rats in both groups (Table 1). Using a linear mixed model and interaction of time and group, we did not find any difference between two groups regarding the slope of epithelial defect decrease (P = 0.521). Log-rank test was run to compare the rate of healing between the two groups (Fig. 1). As it is shown, there was no significant difference in the speed of complete recovery between the two groups. The mean and standard deviation of the two groups in terms of inflammation grade, neovascularization grade, and epithelial thickness are shown in Table 2. No significant difference was observed between the two groups in terms of any of these criteria (Figs. 2, 3, 4). Keratinization was observed in histopathologic examination of one case in the ketamine group and one in the control group (P = 0.83). One case in the ketamine group and one in the control group had a corneal ulcer in the histopathologic examination. One case in the ketamine group had Descemet thickening and folding. There were no pathologic findings or significant difference in number of endothelial cells between case and control group.

**Table 1:** The percentage reduction in size compared to day zero, based on Mann–Whitney test.

Days	Group		Difference	95%		P
	Ketamine	Control		Lower	Upper	
1	42.56 ± 17.83	26.16 ± 23.4	16.41	− 7.52	40.34	0.069
2	68.85 ± 19	60.53 ± 34.21	8.32	− 22.77	39.41	0.796
3	86.25 ± 16.94	75.98 ± 19.09	10.27	− 10.76	31.30	0.427
4	92.57 ± 15.04	95.45 ± 9.09	− 2.88	− 21.82	16.06	0.904
5	98 ± 5.66	92.5 ± 16.77	5.50	− 8.40	19.40	0.642
6	100 ± 0	100 ± 0	0.00			1.000
7	100 ± 0	100 ± 0	0.00			1.000

**Table 2:** Mean and standard deviation of the two groups in terms of inflammation grade, neovascularization grade, presence of keratinization and epithelial thickness.

	Group				P
	Case		Control		
	Mean	Standard deviation	Mean	Standard deviation	
Inflammation grade	1.88	0.83	2.00	0.63	0.726
Neovascularization grade	1.13	1.36	0.33	0.52	0.314
Epithelial thickness	5.71	0.95	5.00	1.41	0.315

## Discussion

The definition of pain according to the International Association for the Study of Pain (IASP) is “an unpleasant sensory and emotional experience associated with actual or potential tissue damage”^14^. Neuropathic pain is caused by nerve fiber lesions or diseases and continues after tissue repair. In this pain, the damaged nervous system transmits ectopic signals to the spinal cord and supra-spinal pain centers^15^. The clinical characteristics of neuropathic pain may be presented in sensory symptoms such as dysesthesia; an unpleasant sensation, hyperalgesia; increased response to painful stimuli and allodynia; creating pain following a non-painful stimulus^16^. Peripheral corneal nerve fiber lesions and aberrant axonal regeneration can create dry-eye syndrome with symptoms such as dysesthesia, hyperalgesia and allodynia. One of the critical factors involved in the development of corneal neuropathic pain is central sensitization, which causes resistance to traditional treatment^17^. The corneal pain system plays a central role in restoring the functional optical tear layer for maintaining healthy vision^18^. There is data showing that excitatory neurotransmitters such as glutamate have a fundamental role in the development and maintenance of neuropathic corneal pain. Glutamate plays its role in a non-competitive block of *N*-methyl-d-aspartate (NMDA) receptors^19^. It was shown that the administration of ketamine, an NMDA antagonist, led to a sustained delay in the improvement of hyperalgesia and allodynia in a rat model with spinal cord lesion^20^. Moreover, systemic and intrathecal administration of ketamine decreased hyperalgesia and chronic pain in experimental models with peripheral nerve fiber lesions^21^. It was found that ketamine has a main role in mitigating neuropathic pain via blocking NMDS receptors and narrowing the influx of calcium ions through the receptor channel. Also, these studies found that ketamine has a critical role in analgesic efficacy in experimental chronic pain model by improving central descending inhibitory pathways^22^. Recently, there is data suggesting a new mechanism for the anti-neuropathic pain role of ketamine by blocking NMDA receptors on the astrocyte membrane of the central nervous system^23^. There is evidence that supports the analgesic effect of ketamine in acute and chronic postoperative pain in clinical trials and experimental studies^9^. In this regard, one study concluded that ketamine lowered the hypersensitivity of damaged nerve fibers at an early stage after surgery in an experimental study^10^. Considering the beneficial effects of ketamine in reducing neuropathic pain, we decided to evaluate the effect of topical ketamine on postoperative chronic ocular pain. Since there was no related research in this regard, it was first necessary to evaluate the safety of topical ketamine on the damaged corneal tissue. Our study showed that topical ketamine did not affect the stage, duration and rate of corneal epithelial defect healing. However, several previous studies identified that systemic administration of ketamine may cause corneal endothelial damage with or without inflammation^24^. The first case report to describe corneal edema after ketamine administration demonstrated this phenomenon through ketamine toxicity to the corneal endothelium with cell loss^25^. Moreover, there is an association between the use of amantadine, an NMDA antagonist, corneal edema and permanent endothelial damage^26^. Similarly, another study showed that memantine by blocking the NMDA receptors led to corneal edema and endothelial damage^27^. In addition, it was found that the administration of a combination of ketamine and xylazine (α2 agonist) reversibly caused corneal edema in an experimental study, which was suppressed by adding yohimbine (an α2 antagonist)^24^. A previous study found that corneal damage in rats after the administration of a mixture of ketamine and xylazine does not occur with each agent alone, and the damage can be minimized through yohimbine. it was also revealed that the phenomenon may be related to agents’ interaction or physiologic consequences of a combination of ketamine and xylazine^28^. However, the mechanism of this phenomenon is still unknown. Some authors hold that tear evaporation and changes in pH that may promote calcium precipitation might have played a role^29^. Another study concluded that corneal lesions after the administration of ketamine–xylasine may be due to persistent drug-induced vasoconstriction of ciliary and iridial vessels as well as prolonged corneal hypoxia and subsequent corneal cell damage^30^. However, all these findings are reported in the experimental model and there have been no reports to date about the association between ketamine and corneal damage in humans. On the other hand, ketamine in these studies was administered systemically and the dose administered in them is much higher than the dose of the drug in topical form usage. However, previous studies have shown that topical administration of some drugs can induce damage to the corneal epithelium. There is evidence that the administration of topical proparacaine and high concentrations of lidocaine can lead to structural changes in endothelial cells of the cornea^31^. Similarly, it was reported that the administration of topical dorzolamide causes corneal endothelial dysfunction especially in subjects with pre-existing endotheliopathy^31^. Nevertheless, no earlier study was found on the effect of topical ketamine on the cornea tissue. In our study, we analyzed the size of the epithelial defect and the rate of reduction in the defect size in the ketamine group compared to the control group. There was no significant difference between the two groups based on epithelial defect size reduction compared to day zero. It was shown that the epithelial defect has healed completely on the sixth day in all rats in both groups. Also, there was no significant difference in the speed of complete recovery between the two groups. Moreover, the eyes of both groups were examined for the amount of inflammation, neovascularization, keratinization, epithelial thickness and Descemet's membrane pathologies. There was no significant difference between both groups based on inflammation grade, neovascularization grade, presence of keratinization, and epithelial thickness. In our study, one case in the ketamine group and one in the control group had a corneal ulcer in the histopathologic examination. It might be related the adverse effect of topical usage of Ketamine, or strongly related to presence epithelial defect and the contamination of the ocular surface. One case in the ketamine group had Descemet thickening and folding. It could be explained by corneal edema. Several reasons like long-term epithelial defect, ocular surface inflammation, and drug toxicity can result in corneal edema. There was no significant difference in corneal epithelial repair between both groups, so this event may not be considered directly related to drug toxicity. However, the future studies with larger sample sizes are required.

One limitation of our study is the small number of rats included. In this study, we only assessed the safety of Ketamine as a topical drop. However, like any other newly introduced drug, phased manner studies should be done by gradually increasing the sample size to investigate the safety and efficacy of a drug before putting the substance to human use.

Finally, it was demonstrated in this study that topical ketamine had no significant adverse effect or no obviously delay on corneal wound healing in rat animal model as compared to control group and that it possibly could be used safely for the management of chronic ocular pain. However, more extensive evaluations in the human model are necessary to determine its safety and efficacy.

## Supplementary Information

Supplementary Information.

## Data Availability

All data generated or analyzed during this study are included in this published article as a supplementary information file.
